# ELSID-Diabetes study-evaluation of a large scale implementation of disease management programmes for patients with type 2 diabetes. Rationale, design and conduct – a study protocol [ISRCTN08471887]

**DOI:** 10.1186/1471-2458-5-99

**Published:** 2005-10-04

**Authors:** Stefanie Joos, Thomas Rosemann, Marc Heiderhoff, Michel Wensing, Sabine Ludt, Jochen Gensichen, Petra Kaufmann-Kolle, Joachim Szecsenyi

**Affiliations:** 1 Department of General Practice and Health Services Research, University of Heidelberg, Voßstr. 2, D-69115 Heidelberg, Germany; 2 Centre for Quality of Care Research, Radboud University Medical Centre Nijmegen, P.O. Box 9101, 6500 HB Nijmegen, The Netherlands; 3 Department of General Practice, University of Frankfurt, Theodor- Stern-Kai 7, D-60590 Frankfurt am Main, Germany; 4 AQUA-Institute for Applied Quality Improvement and Research in Health Care, Weender Landstr. 11, D-37073 Goettingen, Germany

## Abstract

**Background:**

Diabetes model projects in different regions of Germany including interventions such as quality circles, patient education and documentation of medical findings have shown improvements of HbA1c levels, blood pressure and occurrence of hypoglycaemia in before-after studies (without control group). In 2002 the German Ministry of Health defined legal regulations for the introduction of nationwide disease management programs (DMP) to improve the quality of care in chronically ill patients. In April 2003 the first DMP for patients with type 2 diabetes was accredited. The evaluation of the DMP is essential and has been made obligatory in Germany by the Fifth Book of Social Code. The aim of the study is to assess the effectiveness of DMP by example of type 2 diabetes in the primary care setting of two German federal states (Rheinland-Pfalz and Sachsen-Anhalt).

**Methods/Design:**

The study is three-armed: a prospective cluster-randomized comparison of two interventions (DMP 1 and DMP 2) against routine care without DMP as control group. In the DMP group 1 the patients are treated according to the current situation within the German-Diabetes-DMP. The DMP group 2 represents diabetic care within ideally implemented DMP providing additional interventions (e.g. quality circles, outreach visits). According to a sample size calculation a sample size of 200 GPs (each GP including 20 patients) will be required for the comparison of DMP 1 and DMP 2 considering possible drop-outs. For the comparison with routine care 4000 patients identified by diabetic tracer medication and age (> 50 years) will be analyzed.

**Discussion:**

This study will evaluate the effectiveness of the German Diabetes-DMP compared to a Diabetes-DMP providing additional interventions and routine care in the primary care setting of two different German federal states.

## Background

Diabetes is a major and growing health care problem. The number of diabetes patients is expected to increase globally from 135 to 300 million between 1995 and 2025 [1], the vast majority will have type 2 diabetes [2]. The quality of care for diabetic patients in Germany was like in many western countries criticized for more than two decades. The Expert Committee of the Government therefore recommended in 2003 diabetes as a priority area [3]. Epidemiological data from primary care was mostly lacking, although recently published data showed that performance of practices and diabetes control in patients could be better than suggested [4-6]. Quality of care is expected to improve within disease management programs (DMP) by the implementation of evidence-based clinical practice, by means of guidelines, quality circles, educational meetings, outreach visits, patient education and by improving coordination among different health care providers [7,8]. Consequently, clinical guidelines and DMPs for primary care have been developed to improve quality and cost-effectiveness of health care for chronic conditions such as diabetes.

In the reform law of 2002 the German Ministry of Health defined a complicated process for the introduction of DMPs and on 27 February 2003 the Federal Insurance Office accredited the first DMP for type 2 diabetes [9]. Several patient- and provider-oriented interventions within the German-style DMP aiming at decreasing mortality and morbidity of patients with type 2 diabetes reducing micro- and macrovascular complications and increasing quality of life of diabetes patients. The underlying criteria therefore have been developed by the newly formed Coordinating Committee and have gone through a tough process of certification before they have been regulated by law by the Ministry of Health in 2002 [10]. The German DMP structure is tightly linked to financial incentives for the sickness funds, i.e. it is linked with the risk structure compensation (= RSC) which was introduced to compensate for differences in the risk structure of the insured population. [9]. Besides the requirement of a comprehensive documentation and the obligation to provide guideline-oriented healthcare the statutory health insurances are obliged to evaluate DMPs by the Fifth Book of Social Code [9,11].

However, until now valid scientific data showing the effectiveness of the German Diabetes-DMP are missing. Recently published data of diabetes model projects in the region of Nordrhein, Sachsen-Anhalt and Baden-Wuerttemberg show improvements of HbA1c levels, blood pressure and occurrence of hypoglycaemia [12-14]. However, the duration of these model projects was too short to draw conclusions concerning clinical endpoints (i.e. a cardiovascular events, amputation rate) and bias because of the preferred inclusion of highly motivated patients can not be excluded [15].

## Methods/Design

### Aim and design of the study

The aim of the study is to assess the effectiveness of DMP compared to routine care in the primary care setting of two different German federal states (Rheinland-Pfalz and Sachsen-Anhalt). Since DMPs are liable to an implementation process ongoing at the moment which is differing considerably depending on several criteria (e.g. region of Germany, rural/urban area, health insurance) three groups will be observed: a "routine care group without DMP" (= control group; CG), a "DMP real group" (= DMP 1) and a group of patients participating in an (within the scope of the German law regulations) optimally in practices implemented DMP (= DMP 2).

**Figure 1:**
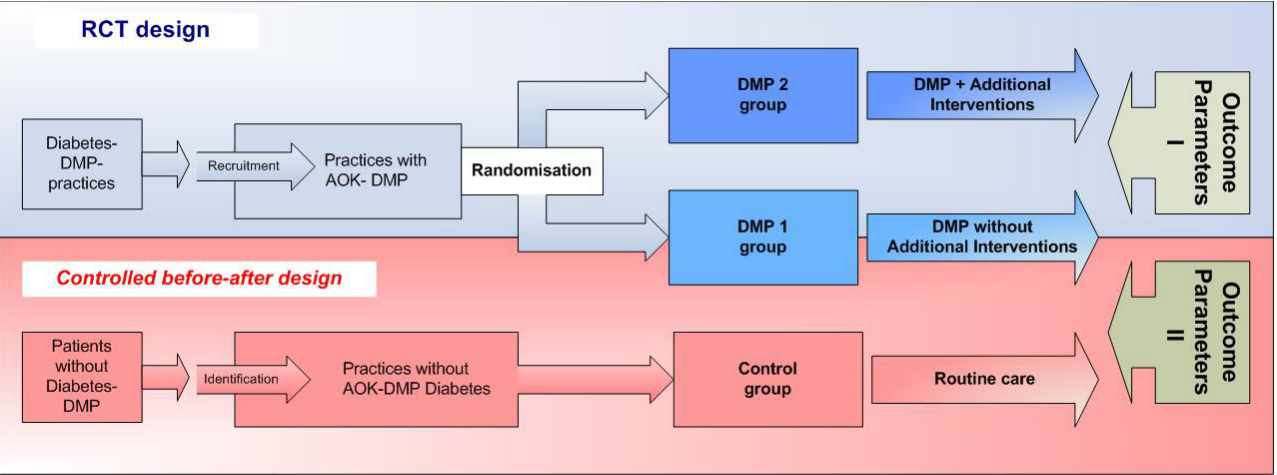
Study design.

The study is designed as a prospective cluster-randomized comparison of the two intervention groups (DMP 1 and DMP 2) against routine care as control group (Fig 1). The cluster randomization was chosen because this has optimal internal validity (absence of confounders) while avoiding contamination of interventions associated with patient randomisation.

### Scientific hypotheses

• The German-Diabetes-DMP (DMP 1) is more effective than routine diabetes care (CG)

• An optimally implemented form of the Diabetes-DMP (DMP 2) is more effective than the German-Diabetes-DMP (DMP 1).

#### Sample size calculation

Sample size calculations for cluster randomized trials differ from sample size calculations for common RCTs requiring a larger number of patients. Based on the main outcome parameters we performed a power calculation with the Cluster Randomization Sample Size Calculator ver.1.02 of the University of Aberdeen on the basis of an ICC of 0,05. Considering possible drop-outs a sample size of 200 GPs will be required (each GP including 20 patients).

#### Selection of participants

500 GPs in Rheinland-Pfalz and 500 GPs in Sachsen-Anhalt participating in the Diabetes-DMP will be recruited by an invitation letter giving information about the study project. From those who will accept to participate, 100 GPs will be randomized in the DMP group 1 and 100 in the DMP group 2. All patients with a type 2 diabetes out of these practices insured at the general regional health funds (*Allgemeine Ortskrankenkassen = AOK*) and participating in the DMP will be included in the study then. Incomplete participation in the interventions will be an exclusion criterion in the DMP group 2.

For the control group 4000 AOK patients out of practices not participating in the DMP will be detected through diabetes tracer medication and age (> 50 years) and will be matched. Participation in a DMP offered by another health fund is an exclusion criterion in the control group.

### Data collection and analysis

All data will be provided by the AOK. Data of the DMP groups are retrieved by the DMP documentation forms which have to be completed routinely every 3 to 6 months during the scheduled DMP visits of participating patients. The information about the tracer medication identifying patients of the control group is also provided by the AOK. The data reporting will follow the CONSORT recommendations for cluster randomized trials [16].

Data analysis will be performed according to the intention-to-treat (ITT) method. For missing data the 'last observation carried forward' -method will be used.

#### Outcome-parameter

Outcome parameters were defined considering best available evidence, legal regulations and feasibility. According to our two scientific hypotheses two groups of outcome parameters were defined: "outcome parameters I" for the comparison of the DMP groups and "outcome parameters II" for the comparison of DMP and routine care. This differentiation of the outcome parameters is essential because in the control group availability of patient data will be limited to process indicators only. The outcome parameters are displayed in Table 1.

**Table 1:** Primary and secondary outcome parameters

**OUTCOME PARAMETERS I**	**OUTCOME PARAMETERS II**
**DMP 1 vs. DMP 2**	**DMP 1 vs. CG**
***Primary outcome parameter***	***Primary outcome parameter***

Proportion of patients achieving target values for HbA1c and RR according to the legal regulations (10)	Proportion of patients with prescriptions for antidiabetic, antihypertensive and lipid-lowering drugs

***Secondary outcome parameters***	***Secondary outcome parameters***

Proportion of patients with prescriptions for antidiabetic, antihypertensive and lipid-lowering drugs	----
Proportion of patients with referrals to ophthalmologists, specialists for diabetology and diabetic feet	Proportion of patients with referrals to ophthalmologists, specialists for diabetology and diabetic feet
Proportion of patients referred to a patient education training for diabetes and hypertension	Proportion of patients referred to a patient education training for diabetes and hypertension
Proportion of patients with severe complications (amputation, dialysis etc.)	Proportion of patients with severe complications (amputation, dialysis etc.)
Proportion of patients with > 2 hospitalizations in the last 6 months	Proportion of patients with > 2 hospitalizations in the last 6 months
Consultation rate	Consultation rate
Days of incapacity to work	Days of incapacity to work
Mean differences of HbA1c, RR, BMI and glomerular filtration rate	---
SCORE risk chart (RR, cholesterol, smoking status, age, gender)	---
Drop out rate from the DMP	---

### Intervention

The minimum requirements for the Diabetes-DMP are regulated by law and defined in the RSAV [10]. According to the legal regulations the intervention in the DMP1 group comprises consultations at 3- or 6-months intervals. During these consultations a detailed diabetes-specific anamnesis and physical examination incl. taking blood pressure and an analysis of HbA1c are carried out. Furthermore agreements are made concerning further treatment, e.g. target values for HbA1c and blood pressure and participation on patient education programs for diabetes or hypertension. All medical findings as well as the current medication have to be documented within structured, standardized documentation sheets at each consultation. If required a referral to a specialist (e.g. ophthalmologist) will be arranged. Furthermore the GPs get a special diabetes-handbook including current, evidence-based information about diabetes therapy. The GPs participating in the DMP1 group will receive no additional intervention.

There are major difficulties introducing new evidence into general practice requiring comprehensive implementation support at different levels (patients, doctors, practice team) [17,18]. Therefore, in the DMP 2 group implementation support at the level of doctors and practice team will be provided. In addition to the clinical interventions at patients level in the DMP group 1 several components aiming at optimizing the implementation process are provided in this group (= implementation interventions) (Table 2).

**Table 2:** Implementation Interventions in DMP 2 group

**Interventions**	**Description**
Interactive quality circle meetings	During these meetings all aspects of evidence based treatment of diabetes in a primary care setting will be discussed (2 × per year).
Educational meetings for medical assistants	During these meetings medical assistants will be supported in finding individual strategies for optimal implementation of the DMP in their practices (2 × per year).
Outreach visits	During these meetings individual problems within the implementation process of the DMP will be discussed with the GP and the assistant team (1 × per year)
Homepage with "best practice" examples	Detailed information for the praxis team about Diabetes-DMP incl. case studies via internet (electronic individual feedback)

The control group represents routine care without DMP. There will be no intervention at all.

### Timeframe of the study

The study team will start to invite GPs at the end of September 2005. After receiving a written declaration of their willingness to participate in the study and to accept random assignment to the different groups GPs will be randomized.

Patient inclusion and pre-data collection will start in 2005. The observation period will be 24 months.

### Description of risks

Since the interventions aim at the evidence based improvement of skills of GPs and practice teams serious risks or undesired effects for patients are not to be expected. There are no specific risks related to the study.

### Patient informed consent

Previous to DMP participation patients already receive written and oral information about the content and extent of the DMP by their treating GPs, i.e. about potential benefits for their health and potential risks. Furthermore, patients are informed that DMP data including medical data will be analyzed. In case of acceptance they have to sign a special DMP participation form.

### Ethical principles and vote of the ethics committee

The study is being conducted in accordance with medical professional codex and the Helsinki Declaration as of 1996 as well as the German Federal Data Security Law (BDSG). DMP participation of patients is voluntary and can be cancelled at any time without provision of reasons and without negative consequences for their future medical care.

The study protocol was approved by the ethics committee of the University of Heidelberg in April 2005 (Approval-Nr. 098-2005). Furthermore the evaluation of the DMP is regulated by law in the Fifth Book of Social Code (§137 f. Abs. 4 SGB V).

### Data security/disclosure of original documents

The patient names and all other confidential information fall under medical confidentiality rules and are treated according to German Federal Data Security Law (BDSG).

Data management will be performed by the AQUA-Institute, Goettingen. All study related data are stored on a protected central server. Only direct members of the internal study team can access the respective files. Intermediate and final reports are stored at the office of the Department of General Practice and Health Services Research at the Heidelberg University Clinic.

## Discussion

There are specific difficulties in evaluating the effectiveness of complex interventions such as disease management programs [19], particularly, in our case that implementation process has already started [15]. At the moment it is not conceivable to which phase of the implementation process the DMP actually has preceded. However, the DMP group 2 represents an ideal implementation of the DMP within the scope of the German law regulation.

To adapt the evaluation design to the real conditions of diabetes care in Germany at the moment, but also to consider the ongoing implementation process we have chosen the design of a prospective randomized-controlled comparison of the two DMP groups embedded in the quasi-experimental design of a controlled before-after study with a blinded control group. The quasi-experimental design is predetermined because allocation to the control group can not be perfomed randomly and because baseline values for patients participating in the DMP have not been documented.

A fully experimental design with a randomized control group was rejected by the authors, because this could create an artificial care situation. That is informing patients and GPs of the control group about the study and probably asking for completing any questionnaires would introduce an enormous bias.

According to the theoretical model for design and evaluation of complex interventions by Campbell et al the presented study can be assigned to phase III and IV (15, 20). However, pilot projects have shown that an observation period of 24 months will be too short to show significant differences in severe clinical endpoints (i.e. amputations, diabetic renal insufficiency, cardio-vascular events). Therefore it was decided to have a combination of HbA1c and blood pressure as primary endpoint in the randomized part of the trail and prescriptions in the quasi-experimental part of the trial.

## Competing interests

The authors declare that they have no competing interests. A contract between the authors and the funder (who might have an interest to show the effectiveness of DMP in this context) ensures that the authors have the full scientific responsiblility and will publish the results in any case.

## Authors' contributions

SJ, TR, JS and MW conceived the study and draft the manuscript. SL, MH, JG and PKK participated in designing the study. All authors read and approved the final version of the manuscript.

## Pre-publication history

The pre-publication history for this paper can be accessed here:


